# Oligomerization and Cell Egress Controlled by Two Microdomains of Canine Distemper Virus Matrix Protein

**DOI:** 10.1128/mSphere.01024-20

**Published:** 2021-04-14

**Authors:** Matthieu Gast, Nicole P. Kadzioch, Doreen Milius, Francesco Origgi, Philippe Plattet

**Affiliations:** a Division of Experimental Clinical Research, DCR-VPH, Vetsuisse Faculty, University of Bern, Bern, Switzerland; b Division of Neurological Sciences, DCR-VPH, Vetsuisse Faculty, University of Bern, Bern, Switzerland; c Graduate School for Cellular and Biomedical Sciences, University of Bern, Bern, Switzerland; d Institute of Science and Technology (IST), Bioimaging Facility, Klosterneuburg, Austria; e Centre for Fish and Wildlife Health (FIWI), Department of Infectious Diseases and Pathobiology, Vetsuisse Faculty, University of Bern, Bern, Switzerland; University of Michigan-Ann Arbor

**Keywords:** morbillivirus cell exit, matrix protein, M-M interaction, VLPs production, dynamics at the cell surface

## Abstract

The multimeric matrix (M) protein of clinically relevant paramyxoviruses orchestrates assembly and budding activity of viral particles at the plasma membrane (PM). We identified within the canine distemper virus (CDV) M protein two microdomains, potentially assuming α-helix structures, which are essential for membrane budding activity. Remarkably, while two rationally designed microdomain M mutants (E89R, microdomain 1 and L239D, microdomain 2) preserved proper folding, dimerization, interaction with the nucleocapsid protein, localization at and deformation of the PM, the virus-like particle formation, as well as production of infectious virions (as monitored using a membrane budding-complementation system), were, in sharp contrast, strongly impaired. Of major importance, raster image correlation spectroscopy (RICS) revealed that both microdomains contributed to finely tune M protein mobility specifically at the PM. Collectively, our data highlighted the cornerstone membrane budding-priming activity of two spatially discrete M microdomains, potentially by coordinating the assembly of productive higher oligomers at the PM.

**IMPORTANCE** Despite the availability of efficient vaccines, morbilliviruses (e.g., canine distemper virus [CDV] and measles virus [MeV]) still cause major health impairments. Although antivirals may support vaccination campaigns, approved inhibitors are to date still lacking. Targeting late stages of the viral life cycle (i.e., the cell exit system) represents a viable option to potentially counteract morbilliviral infections. The matrix (M) protein of morbillivirus is a major contributor to membrane budding activity and is assumed to assemble into dimers that further associate to form higher oligomers. Here, we rationally engineered M protein variants with modifications in two microdomains that potentially locate at dimer-dimer interfaces. Our results spotlight the cornerstone impact of both microdomains in membrane budding activity and further suggest a role of finely tuned high-order oligomer formation in regulating late stages of cell exit. Collectively, our findings highlight two microdomains in the morbilliviral M protein as novel attractive targets for drug design.

## INTRODUCTION

Canine distemper virus (CDV) is a nonsegmented, negative-stranded, enveloped RNA virus belonging to the genus *Morbillivirus* within the *Paramyxoviridae* family (1). CDV is closely related to the rinderpest and peste-des-petits-ruminants viruses, which infect large and small ruminants, and to the highly contagious measles virus (MeV) that infects humans and nonhuman primates (2). Canine distemper, also known as Carre’s disease (3), is a highly contagious viral infection affecting dogs and a broad range of terrestrial animals among wildlife and domesticated species (4, 5). Although a very efficient vaccine is available for dogs, vaccine failures or fatal outcomes associated with vaccinations have been reported in other species (e.g., black-footed ferrets), which warranted the development of safer vaccines and/or therapeutics to better control ongoing epidemics (6).

CDV-associated disease is characterized by a multisystemic infection, frequently affecting the gastrointestinal and respiratory systems, along with lymphoid organ depletion (7) and a possible progression to the central nervous system (CNS) (7, 8). Morbilliviruses use at least two cellular receptors to invade their hosts: signaling lymphocyte activation molecule (SLAM or CD150) and nectin-4. While SLAM is used as the primary receptor to infect some immune cells (9), nectin-4 is employed by the virus to spread in epithelial cells (10, 11). The putative receptor(s) expressed in the central nervous system (CNS) remains undetermined.

The CDV genome encodes eight proteins divided into two groups: six structural proteins (N, P, M, F, H, and L) and two accessory proteins (V and C). The nucleocapsid (N) protein encapsidates the genomic RNA into a helical assembly (8) on which the viral polymerase (*P+L*) gains access to form the ribonucleoprotein (RNP) complex (12). The CDV envelope comprises two transmembrane glycoproteins, the attachment and fusion proteins (H and F, respectively), mostly involved in the mechanism of cell entry. Finally, a membrane-associated protein called matrix (M) protein is responsible for the virion assembly and budding processes from the host cell plasma membrane (13).

The M protein is assumed to interact with the C-terminal domain of the N protein, as well as the cytoplasmic tails of the surface H and F glycoproteins, thereby orchestrating the viral assembly process (14–18). In addition, M collects beneath the host cell plasma membrane and triggers the formation of membrane protrusions, which may act as platforms from which virions can bud into the extracellular spaces (16, 19–24). The final stages mentioned above are further regulated by M interaction with cellular actin filaments, although some variations in the mechanisms promoting M-driven membrane budding activity might be strain dependent (16, 20, 25). Interestingly, the M proteins of several members of the families *Paramyxoviridae* and *Pneumoviridae* harbor the intrinsic ability to drive the formation of virus-like particles (VLPs) in the presence or absence of other viral components (19, 26–31). To promote the release of VLPs and/or virions, specific microdomains of the M protein (i.e., late domains) are proposed to contribute to the molecular mechanism underlying membrane budding activity of many enveloped viruses (e.g., HIV-1 and Ebola virus). These domains, which vary among viruses (e.g., PPxY, P[T/S]AP, or YxxL) recruit and interact with cellular proteins of the endosomal sorting complex required for transport (ESCRT), leading to the release of virus particles (32). Interestingly, in contrast to other members of the *Mononegavirales* and other paramyxoviruses, the morbillivirus particle scission process seems to occur in an ESCRT-independent manner (31, 33, 34). Moreover, MeV budding can also occur in the absence of the M protein, but at very low levels, and the nature of the released progeny virions remains uncertain (35). Interestingly, a previous study demonstrated that a single point mutation (V101A) within the M protein of MeV (MeV-M) was sufficient to considerably reduce the release of viral particles into the supernatant (19). Additional studies revealed a role of the amino acid at position 89 of MeV-M as an important determinant for adaptation and growth of the virus in Vero cells (36, 37). Indeed, while the substitutions E89G and E89K allowed the virus to adapt to Vero cells, the mutant M-E89K enabled MeV to more efficiently infect cotton rats and affect virus replication in human peripheral blood mononuclear cells (PBMCs) (37–40). Finally, M-driven membrane budding may also rely on its capacity to associate with cellular membranes, as well as to self-assemble into stable dimers, which in turn may form proper building blocks to assemble high-order oligomers (6, 14, 19, 21, 22).

No structural data for any morbillivirus M protein are currently available. However, high-resolution atomic structures for the M protein of related viruses (Newcastle disease virus [NDV], human metapneumovirus [hMPV], and respiratory syncytial virus [RSV]) were determined. These crystallographic studies revealed high structural conserved motifs, including (i) one M protomer composed of amino-terminal and carboxyl-terminal domains (NTD and CTD, respectively) linked by a putative flexible linker, and (ii) two protomers assemble into dimers via a large dimeric binding interface in an antiparallel “head-to-tail” fashion (6, 41–43). Further investigations by electron microscopy (EM) revealed that M can form high-order oligomers within the viral particle (41, 42, 44, 45). Two putative surface-exposed α-helices, namely, α2 and α9, located within the NTD and CTD, respectively, and on opposite sides of the dimeric binding interface, were proposed to be involved in the formation of high-order M oligomeric arrays (6). Interestingly, it has been suggested that the angle generated between two M dimers, as well as the avidity of the dimeric interface, would contribute to the membrane curvature required for efficient viral budding (6).

In the present study, we aimed to gain further mechanistic insights into the molecular dynamics promoting efficient membrane budding activity. More specifically, we investigated the role of two spatially discrete M microdomains (putatively folding into α-helices) in the formation of high-order oligomeric arrays and their potential correlation with the viral egress process. Our findings suggest that both microdomains critically contribute to the formation of productive oligomers under the plasma membrane, which in turn are pivotal in driving efficient membrane budding activity.

## RESULTS

### Single mutations in potentially surface-exposed α-helices of the M protein did not alter dimerization.

**FIG 1:**
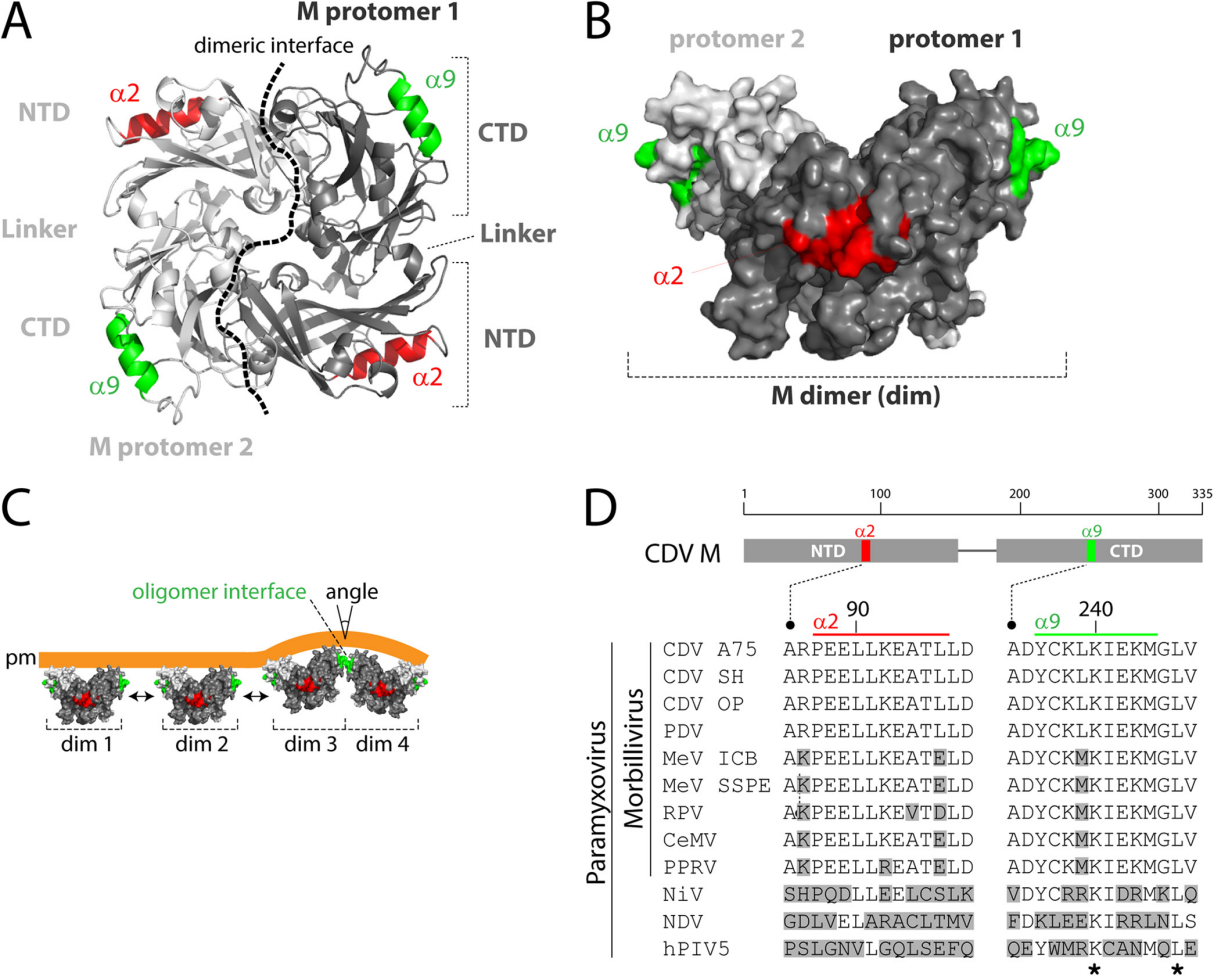
Structural model of CDV M and the derived putative oligomerization process. (A) NDV M-based (PDB code 4G1G) structural homology model of CDV M dimer. The two major microdomains of both protomers are colored in red (α2-helix) and green (α9-helix). The protomer-protomer binding interface is represented with a dashed black line. (B) Three-dimensional representation of a CDV M dimer with the putative α-helices colored in red (α2) and green (α9). The view illustrates the potentially surface-exposed α9-helix. (C) Model of M-mediated membrane budding activity. Bidirectional M dimer assembly (dim 1 to dim 4) may generate angles at the dimer-dimer binding interfaces, which in turn may contribute to the induction of lipid curvatures and ensuing viral budding. (D) Sequence alignment of M proteins of various paramyxoviruses. The M protein main domains and regions targeted for mutagenesis (highlighted in red or green) are schematically represented at the top. Accession numbers (GenBank or NCBI) for the virus sequences are as follows: CDV A75/17 (CDV A75), AAD49701; CDV Snyder Hill (CDV SH), AFC40215; CDV Onderstepoort (CDV OP), NP_047204; phocine distemper virus (PDV), AGL33552; MeV IC-B (MeV ICB), BAM36454; MeV genotype B3, an SSPE strain (MeV SSPE), ALL29057; rinderpest virus (RPV), CAA53779; cetacean morbillivirus (CeMV), AEO51054; peste-des-petits-ruminants virus (PPRV), ACQ44669; Nipah virus (NiV), NP_112025; Newcastle disease virus (NDV), AEZ36128; and human parainfluenza virus 5 (hPIV5), ABC75775. The stars represent amino acids conserved throughout members of the subfamily. Amino acids differing from the reference CDV M sequence (strain A75/17) are shown in gray boxes.

Structural studies provided evidence of M oligomeric network formation underneath both the plasma membrane and the viral envelope (41, 42, 44, 45). However, the low resolution prevented the identification of microdomains that may support the formation of such large M oligomeric arrays. Furthermore, although the angle generated at the putative M dimer-dimer interface is suggested to contribute to lipid curvature and the ensuing plasma membrane budding activity (Fig. 1C), functional evidence is entirely lacking (6).

Hence, to gain mechanistic insights into M functions and, more precisely, the potential correlation between two putative surface-exposed α-helices (α2 and α9, hypothesized to be involved in M dimer-dimer contacts) and the inherent ability of M to bud from the plasma membrane, we generated a series of conservative and nonconservative single M mutants (derived from the neurovirulent A75/17 CDV strain) (Table 1). The positions of the mutations were selected based on a previously generated homology 3D-structural model of M dimers (Fig. 1A and B). In this model, α2 and α9 map in the NTD and CTD, respectively, and both microdomains locate opposite to the large M dimeric interface. Interestingly, residues constituting the two helices are well conserved among members of the genus *Morbillivirus*, consistent with a potential relevant role of these microdomains in M protein activity (Fig. 1D). Note that we conducted a more thorough mutational analysis of the glutamic acid residue at position 89 (Table 1), given that this amino acid was reported to influence MeV replication both *in vitro* and *in vivo* (36–40). Overall, we intended in this study to generate M mutants exclusively deficient in triggering late stages of the viral life cycle (i.e., membrane budding) while preserving other functions mostly unaltered (i.e., folding, intracellular transport-competence, interaction with the nucleocapsid protein [N], and localization with the plasma membrane).

**TABLE 1:** Summary of properties of M-wt and derivative mutants^*a*^

M mutation	Dimerization capacity (>70%)	Intracellular localization	Interaction with N	Drives membrane deformation	VLP production (>70%)
M-wt					
None	Yes	PM	Yes	Yes	Yes
α2 mutants					
P87A	Yes	PM	ND	ND	Yes
K92E	Yes	PM	ND	ND	**No**
A94S	Yes	PM	ND	ND	**No**
L96N	Yes	PM	ND	ND	**No**
E89D	Yes	PM	Yes	Yes	Yes
E89G	**No**	**Cytosol**	ND	ND	**No**
E89H	Yes	PM	ND	ND	**No**
E89K	Yes	PM	ND	ND	**No**
E89R	Yes	PM	Yes	Yes	**No**
E89Y	Yes	**Cytosol**	ND	ND	**No**
α9 mutants					
D235N	Yes	PM	Yes	Yes	Yes
Y236F	Yes	PM	Yes	Yes	**No**
K238E	**No**	**Cytosol**	ND	ND	**No**
L239D	Yes	PM	Yes	Yes	**No**
K240Q	Yes	PM	ND	ND	**No**
E242Q	Yes	PM	ND	ND	Yes
K243E	Yes	PM	ND	ND	**No**
G245P	**No**	**Cytosol**	**No**	**No**	**No**

a ND, not determined; PM, plasma membrane. Boldface type indicates a difference from wild type.

**FIG 2:**
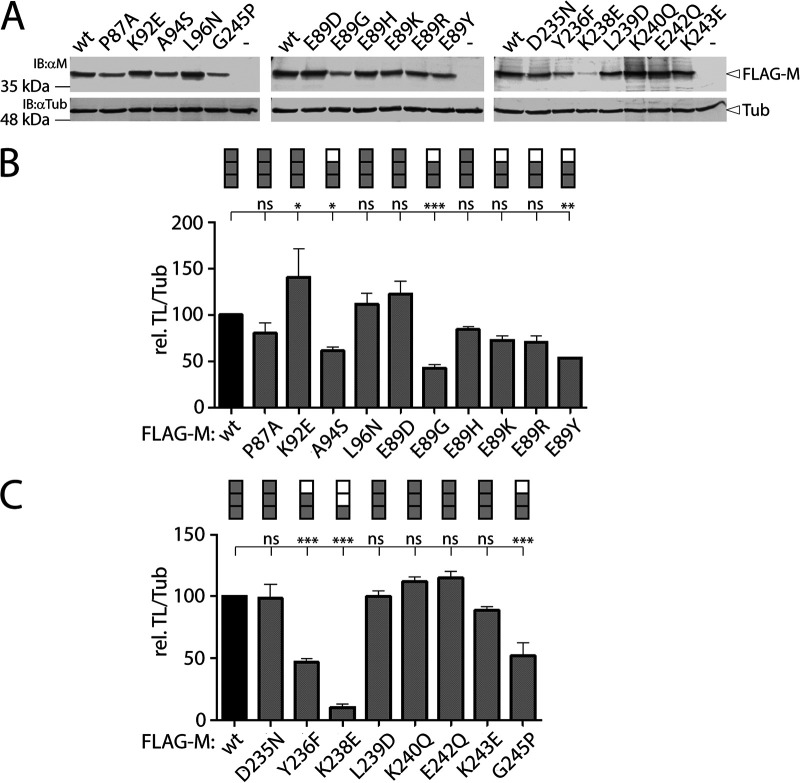
Assessment of the various M protein concentrations. (A) Assessment of the FLAG-tagged M protein constructs concentration by Western blotting analyses. The antibodies used and the position of the designated proteins are indicated. As a loading control, tubulin was detected using a monoclonal anti-tubulin antibody (αTub). (B and C) Quantitative determination of the various M protein concentration profiles (FLAG-M/Tub). Band intensities were recorded with the VILBER software and scored as follow (normalized to M-wt): 0 to 40%, 1 gray box; 41 to 80%, 2 gray boxes; 81 to 140%, 3 gray boxes. Means and standard deviations (SD) of data from three independent experiments normalized to wt are shown. Statistical significance was determined using one-way ANOVA followed by Tukey test using GraphPad Prism 8 to analyze differences between wild-type and mutant means; ***, *P* ≤ 0.001; **, *P* ≤ 0.01; *, *P* ≤ 0.05; ns, not significant.

With the goal to identify such specific M mutants, we first determined the impact of the various mutations on the M proteins' concentration in the cells (Fig. 2). Upon transfection of HEK-293T cells with the various expression plasmids, total proteins were extracted at 48 h posttransfection and submitted to Western blot analyses (Fig. 2A). Overall, while most of the M mutants preserved wild-type (wt)-like intracellular protein concentrations, the M-E89G (α2 mutant) and K238E (α9 mutant) mutants displayed the most drastic deficiencies (<50% compared to M-wt) (Fig. 2B and C). We also arbitrarily scored the protein concentration levels of all mutants (0 to 40%: 1 gray box; 41 to 80%: 2 gray boxes; 81 to 140%: 3 gray boxes; Fig. 2B and C).

**FIG 3:**
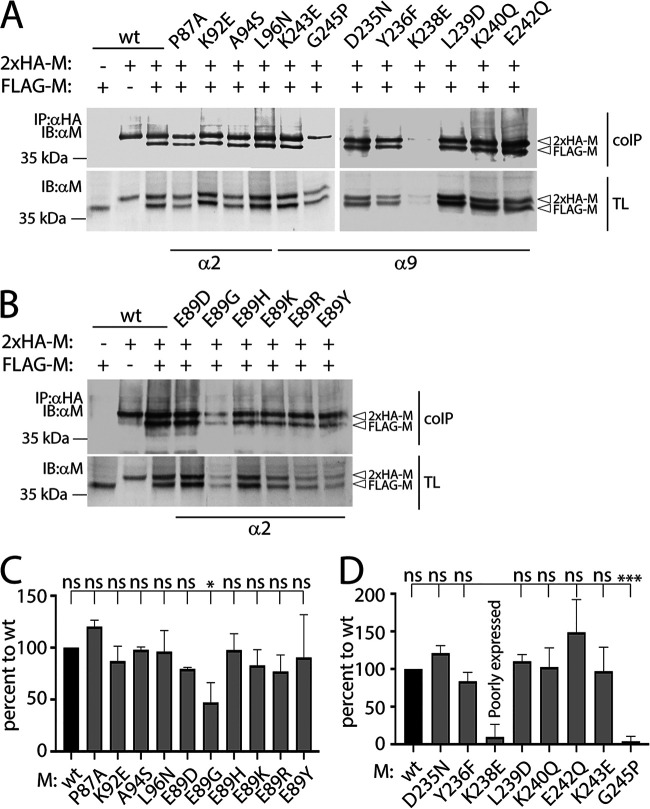
Assessment of the various M protein self-assembly efficiencies. (A and B) Self-assembly efficiencies of the indicated M protein constructs expressed in HEK-293T cells were assessed by coimmunoprecipitation (coIP) and Western blot analysis. The antibodies employed for the immunoprecipitation (IP) and immunoblotting (IB), as well as the position of the FLAG- and 2×HA-tagged M proteins, are indicated. α2/9, mutations created in the putative alpha helices 2 and 9, respectively; TL, total cell lysates. (C and D) CoIP efficiency index of the different M constructs (see the Materials and Methods). Intensities of the bands were quantified using the VILBER software. Means and SD of data from three independent experiments normalized to wt are shown. Statistical significance was determined using one-way ANOVA followed by Tukey test using GraphPad Prism 8 to analyze differences between wild-type and mutant means; ***, *P* ≤ 0.001; *, *P* ≤ 0.05; ns, not significant.

We next investigated whether the selected mutations affected protein folding. Because we lack conformational antibodies to probe for proper CDV M folding, we carried out coimmunoprecipitation (coIP) experiments, assuming that misfolded M proteins may in turn exhibit impairments in dimer formation. To facilitate the distinction between immunoprecipitated (IP) and coIP M proteins, we expressed in HEK-293T cells two M variants that were modified to harbor tags of different size (2×HA-longlinker-M [2×HA-M] and FLAG-linker-M [FLAG-M]). CoIP experiments revealed that, except for M-E89G, all other α2 M mutants maintained their ability to form dimers, which argued for overall proper protein folding (Fig. 3A and B). However, as mentioned above, the intracellular protein concentration of M-E89G was reduced, which suggested defects in the folding capacity of this mutant, in turn translating into improper expression/stability and self-assembly (Fig. 3B). Regarding the α9 M mutants, two out of eight (M-K238E and M-G245P) failed to efficiently assemble into dimers (Fig. 3A). This result was also not surprising. Consistently, in our 3D-structural homology model, the lysine residue at position 238 was found mostly buried inside the protein, thereby suggesting a critical role in protein folding and expression/stability. In addition, we also replaced the glycine residue at position 245 with a proline (G245P) in order to locally affect the α9-helix and eventually prevent oligomerization. Remarkably, coIP analyses confirmed that M-G245P lost dimerization capacity (Fig. 3A), while the intracellular protein concentration of this mutant remained moderate (∼50% compared to M-wt) (Fig. 2A and C).

We additionally calculated the coIP efficiency index, which was determined as the ratio of coIP to IP M signals divided by the ratio of total cell lysates (TL; obtained in Western blot analyses) and normalized to M-wt conditions. Overall, except the mutant M-E89G, all α2 M mutants preserved potent capacity to self-assemble (>70%) (Fig. 3A to C). Conversely, and consistent with the coIP analysis results, the α9 M mutants K238E and G245P showed a dramatically reduced coIP efficiency index compared to the wt (9.7% and 3.8%, respectively; Fig. 3D), whereas all other α9 M mutants had unaffected self-assembly capacities (Fig. 3D).

In summary, this series of experiments indicated that single amino acid substitutions within the α2/9 M microdomains did not impair the intracellular protein concentration and self-assembly (except for M-E89G, M-K238E, and M-G245P), which in turn argues for proper folding of the rationally engineered protein variants. Finally, because our coIP experiments could not distinguish between dimerization and high oligomeric assembly, M mutants exhibiting proper coIP efficiency may be characterized by impaired, or irregular, M oligomeric network formation.

### Intracellular localization of α2/9 M mutants.

**FIG 4:**
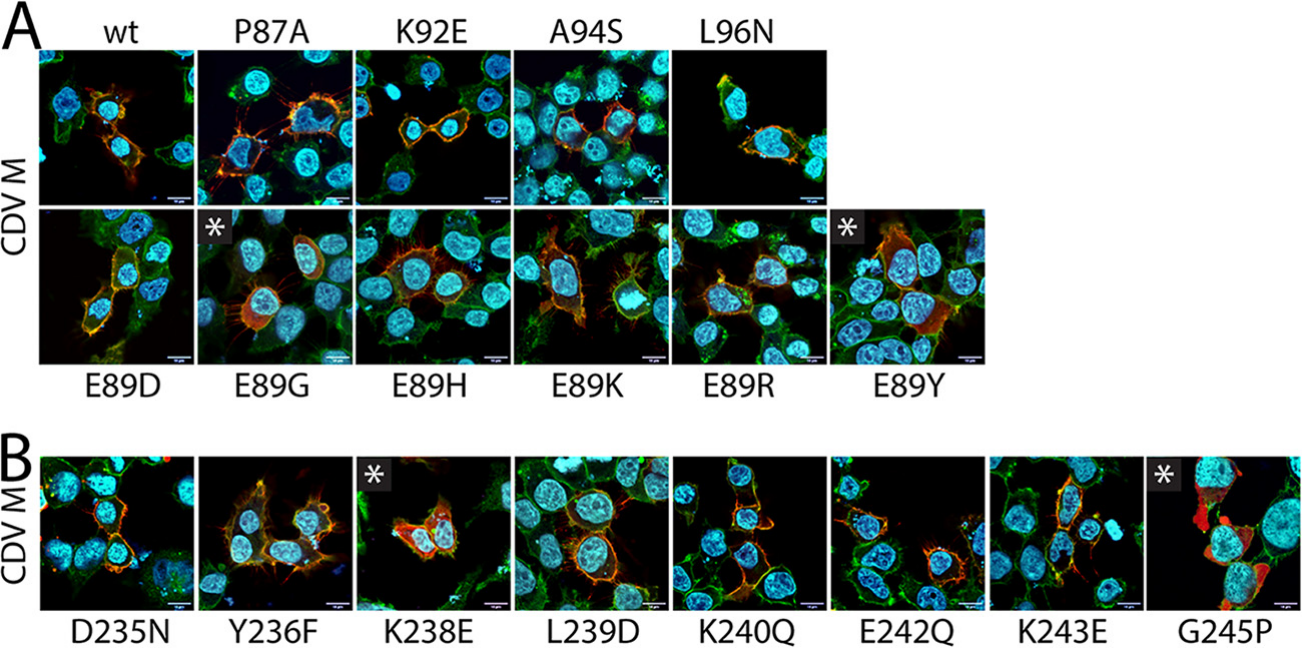
Cellular localization of diverse CDV M constructs. The cellular localization of FLAG-tagged M-wt proteins and derivative α2 mutants (A) and α9 mutants (B), expressed in HEK-293T cells, was investigated by immunofluorescence analyses. Images were captured by laser scanning confocal microscopy using an Olympus FV3000 with FluoView FV3000 system software. FLAG-tagged M proteins were stained in red, the plasma membranes and the nuclei were counterstained in green and blue, respectively. *, cytosolic localization of M.

To determine the impact of the mutated α2/9 microdomains in the cellular localization of CDV M, immunofluorescence (IF) analyses were performed on HEK-293T cells expressing FLAG-tagged M variants. This allowed us to detect the various mutated M proteins using an anti-FLAG monoclonal antibody (MAb) and independently of their possible (even local) modified conformations. Additionally, the cell periphery was highlighted by monitoring the presence of the plasma membrane (PM)-residing alpha 1 subunit of a sodium-potassium pump. As expected, at 24 h posttransfection the M-wt protein localized at the cell periphery (Fig. 4A). While α9 M mutants that preserved efficient coIP efficiency displayed a wt-like phenotype, the two M mutants unable to form dimers (M-K238E and M-G245P) exhibited a cytosolic localization phenotype (Fig. 4B). All the α2 M mutants except M-E89G and M-E89Y properly reached the plasma membrane (Fig. 4A). Both mutants showed (direct or indirect) defects in intracellular trafficking.

Taken together, our results reveal that modifying the α2/9 microdomains of the CDV M protein with single point mutations does not significantly affect PM targeting of the derivative M mutants (except for M-K238E, M-G245P, and M-E89G/Y). Because we aimed at identifying M mutants with proper expression/folding capacity and preserved PM localization, but with defects in membrane budding activity, the phenotype of the latter four mutants was not further investigated. However, the variant M-G245P was employed as negative control for all following experiments.

### M α2/9 mutants with unaffected PM targeting also preserved interaction with the nucleocapsid protein.

Although the above findings demonstrated that most of the generated α2/9 M mutants preserved intact intracellular protein concentration, dimerization, and PM-targeting ability, we wished to further investigate the impact of the substitutions on the inherent capacity of CDV M to interact with the nucleocapsid (N) protein (14, 19, 31). To this aim, we recorded by IF analysis the ability of some selected M proteins to relocate the N protein to the cell periphery. In the absence of M, the N protein is uniformly distributed throughout the cytoplasm, whereas, in its presence, N substantially relocates to the cell periphery (19).

**FIG 5:**
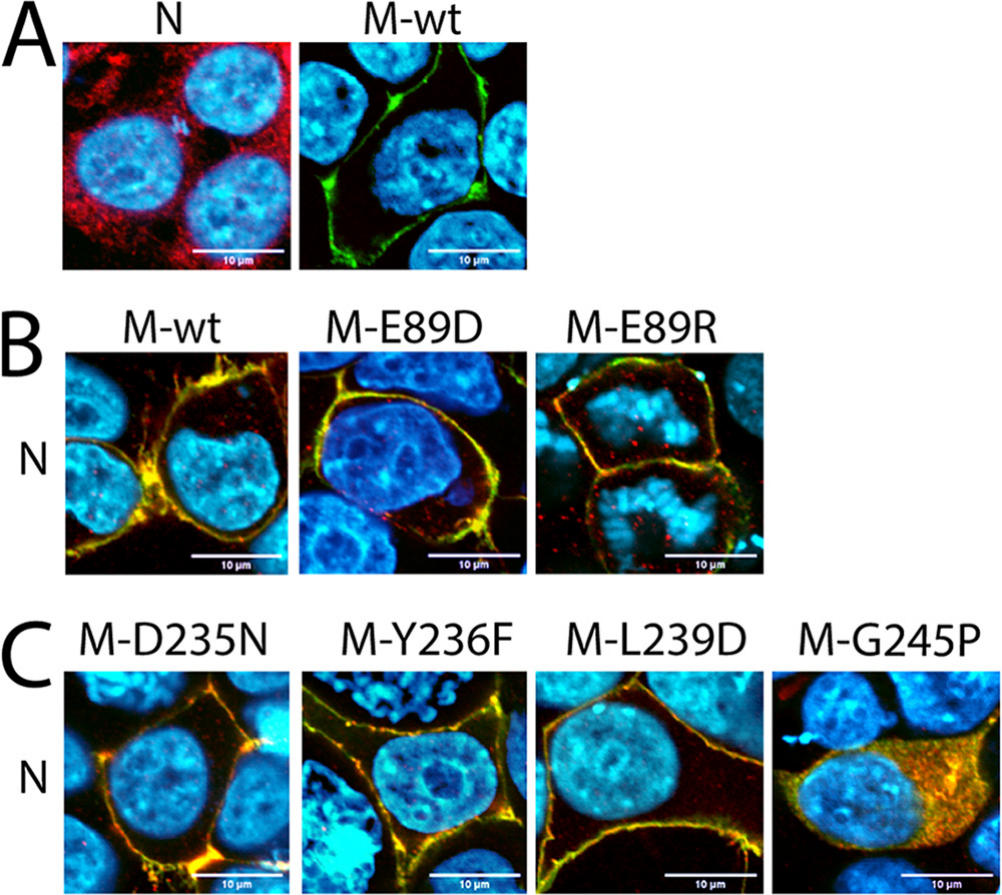
Influence of the M microdomains in cellular N-relocalization. (A to C) HEK-293T cells were transfected with plasmid DNA encoding HA-tagged N proteins alone or in combination with plasmid DNA encoding FLAG-tagged M. Cellular localizations were determined by immunofluorescence analyses. Images were captured by a laser scanning confocal microscope (Olympus FV3000 with FluoView FV3000 system software). N proteins were counterstained in red, M proteins in green, and the nuclei in blue.

To investigate M-N interactions, we subjected the constructs to IF analysis. As expected, while analyses performed on cells expressing N alone indicated a diffuse cytoplasmic accumulation, a clear cell periphery staining was recorded when M was expressed in the absence of N (Fig. 5A). Strikingly, when N and M were coexpressed in the same cells, N was efficiently transported to the cell periphery and clearly colocalized with M (Fig. 5B). While the M mutants E89D, E89R, D235N, Y236F, and L239D exhibited a wt-like phenotype, M-G245P lost PM targeting and lacked N relocalization (Fig. 5B and C).

Collectively, these data further support the notion that substituting single amino acids within the putative α2/9-helices of CDV M does not dramatically affect protein folding, dimerization, intracellular transport competence, and interaction with the CDV N protein.

### M α2/9 mutants efficiently drive membrane deformation.

**FIG 6:**
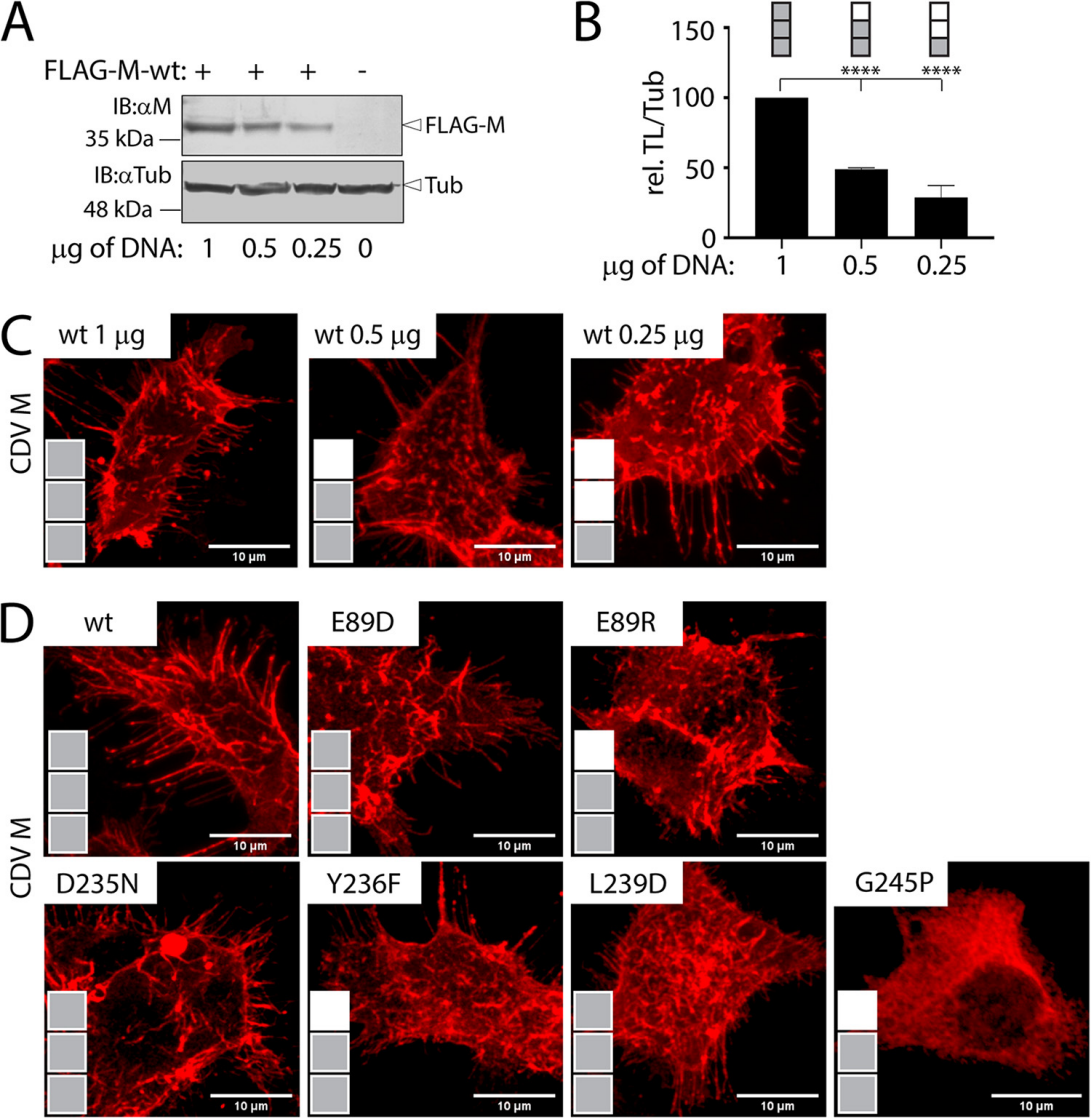
Investigation of plasma membrane deformation triggering by selected M proteins. (A) Assessment of the FLAG-tagged M-wt protein concentration by Western blotting analysis. The antibodies used and the position of the designated proteins are indicated. As a loading control, tubulin was detected using a monoclonal anti-tubulin antibody (αTub). (B) Quantitative determination of M-wt protein concentration profiles (FLAG-M/Tub). Band intensities were recorded with the VILBER software and scored as follow (normalized to 1 μg of transfected DNA plasmid): 0 to 40%, 1 gray box; 41 to 80%, 2 gray boxes; 81 to 140%, 3 gray boxes. (C and D) Immunofluorescence analyses performed from HEK-293T cells expressing M-wt (C) or selected M protein variants (D). Multiple z-stack images were acquired from cells expressing the indicated M proteins by laser scanning confocal microscopy (Olympus FV3000 with FluoView FV3000 system software) and merged. Means and SD of data from three independent experiments normalized to the reference (1 μg of transfected DNA plasmid) are shown. Statistical significance was determined using one-way ANOVA followed by Tukey test using GraphPad Prism 8 to analyze differences between wild-type and mutant means; ****, *P* ≤ 0.0001.

Paramyxoviruses and pneumoviruses can trigger plasma membrane deformation and in turn may employ such structures as a platform for viral budding and cell egress. Accordingly, we determined the capacity of M-wt and selected α2/9 M mutants to drive membrane deformation upon expression in HEK-293T cells. To clearly highlight such membrane structures, an optimal number of successive z-stacks of M-expressing cells were recorded by confocal microscopy and merged. Since differences in the intracellular protein concentration of the various M proteins may influence membrane deformation, we initially transfected increasing amount of plasmid DNA encoding M-wt. Upon Western blot analysis, various levels of M-wt protein concentration were scored as determined above for the mutants (0 to 40%: 1 gray box; 41 to 80%: 2 gray boxes; 81 to 140%: 3 gray boxes; Fig. 2B and C) (Fig. 6A and B). Figure 6C illustrates that, regardless of the protein concentrations, M-wt efficiently triggered membrane deformation, as shown by the production of long cell protrusions in all three conditions. Conversely, in M-G245P-expressing cells, no cell protrusion could be detected, as expected from a mutant that lost its capacity to efficiently reach the PM. Strikingly, all other tested mutants potently triggered membrane deformation (Fig. 6D).

In summary, this series of experiments indicates that M mutants with modified α2/9 microdomains preserve their ability to drive membrane deformation.

### Intact α2/9 microdomains are required to enable M proteins to efficiently release virus-like particles.

**FIG 7:**
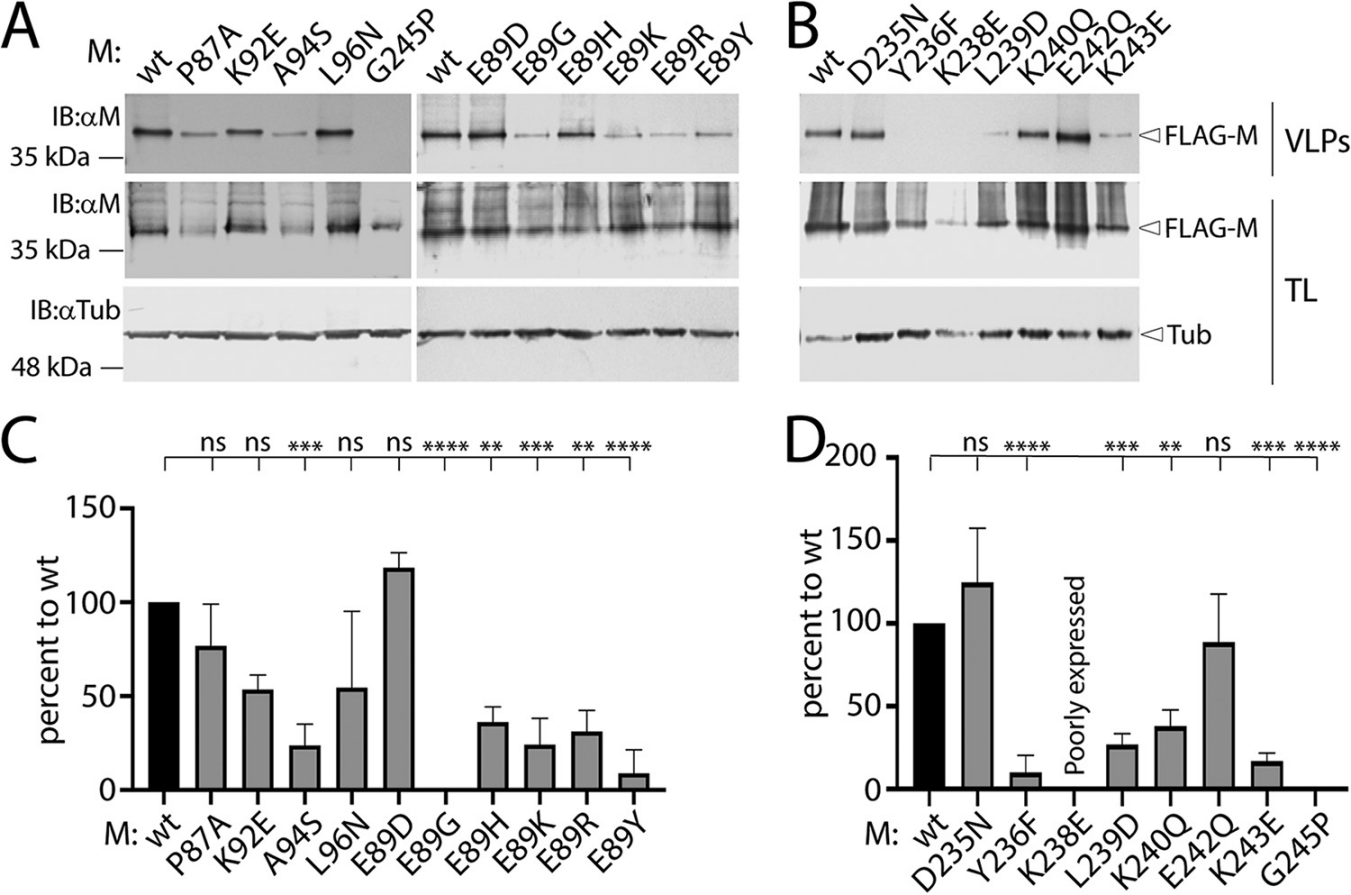
Analyses of VLPs induced by the various M proteins. (A and B) Virus-like-particles (VLPs) were harvested from the supernatants of M-transfected HEK-293T cells (48 h posttransfection). Collected VLPs and corresponding total cell lysates (TL) were subjected to Western blot analysis, where M antigenic material was detected using a polyclonal anti-CDV M antibody (αM). As a loading control, tubulin was detected using a monoclonal anti-tubulin antibody (αTub). (C and D) Quantitative assessment of the VLP efficiency index (see the Materials and Methods). The intensities of the bands were quantified using the VILBER software. Means and SD of data of three independent experiments normalized to wt are shown. Statistical significance was determined using one-way ANOVA followed by Tukey test using GraphPad Prism 8; ****, *P* ≤ 0.0001; ***, *P* ≤ 0.001; **, *P* ≤ 0.01; ns, not significant.

It is well known that matrix proteins of paramyxoviruses and pneumoviruses contribute to the release of virus-like particles (VLPs) even in the absence of any other viral components. To investigate the budding efficiency of CDV M-wt and derivative mutants, the cell supernatants of HEK-293T cells were harvested at 48 h posttransfection. Supernatants were submitted to Western blot analysis, as previously performed (6). VLPs were also subjected to Western blot analysis and the VLP efficiency index was calculated as the ratio between M proteins found in VLPs versus those extracted from total cell lysates. When normalized to M-wt, all but M mutants P87A, E89D, D235N, and E242Q (which had >70% of wt) exhibited a defect in VLPs formation (levels ≤70%) (Fig. 7A to D). While this was expected for M mutants E89G, E89Y, K238E, and G245P (with defective intracellular protein concentration profiles and/or intracellular transport competence), the other mutants (M-K92E, M-A94S, M-L96N, M-E89H, M-E89K, M-E89R, M-Y236F, M-L239D, M-K240Q, and M-K243E) did not bud efficiently despite proper expression, dimer formation, localization at the PM, interaction with N, and triggering of the production of long cell protrusions.

Altogether, these results indicate that introducing mutations at specific positions within the α2/9 microdomains of CDV M could impact VLP production, thereby providing the first insights into the key role of both regions in the cell exit process. These data additionally suggest a prominent impact of the putative α9-helix secondary to the mutations occurring in this microdomain, which more substantially affected the budding capacity of the derivative M proteins.

### Viruses harboring selected substitutions within the α2/9 M microdomains lacked proper viral infectivity.

**FIG 8:**
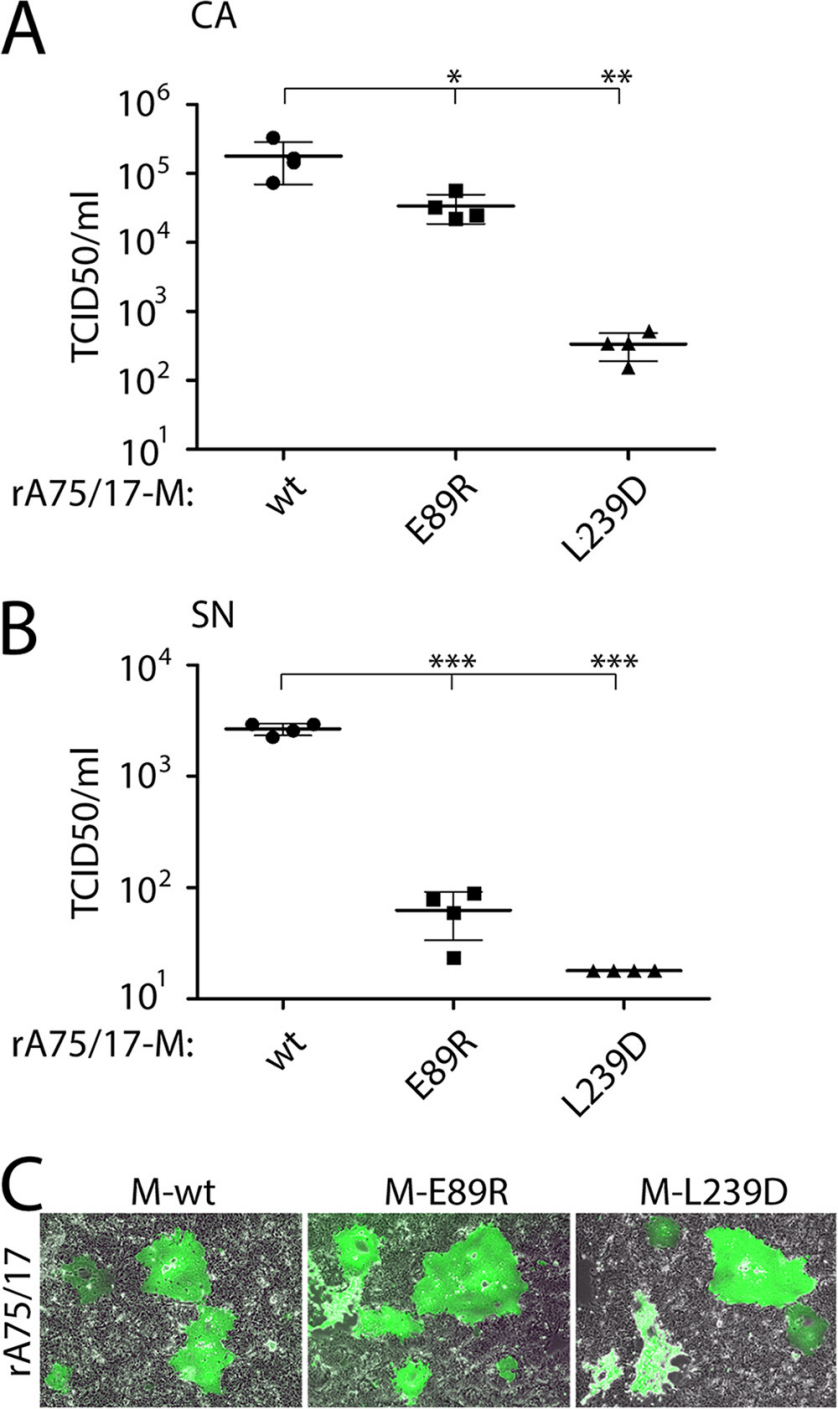
Analysis of infectious virus production by recombinant viruses expressing standard and mutated M proteins. (A and B) Vero-cSLAM cells were infected with VSV-G-complemented rA75/17 viruses expressing wt, E89R, or L239D M proteins for 3 days. Viral titers (TCID_50_) of cell-associated (CA) (A) and cell-free particles in the supernatant (SN) (B) were calculated according to the Spearman & Kärber method. (C) Pictures of representative fields of view were acquired using an Invitrogen EVOS M5000 microscope. Means and SD of data of four independent experiments are shown. Statistical significance was determined using one-way ANOVA followed by Tukey test using GraphPad Prism 8 to analyze differences between wild-type and mutant means; ***, *P* ≤ 0.001; **, *P* ≤ 0.01; *, *P* ≤ 0.05.

We engineered two recombinant viruses harboring a selected single mutation in both presumptive helices (M-E89R in α2 and M-L239D in α9) in order to investigate the impact of such M mutants in a more biologically relevant context (referred to as rA75/17-M-E89R and rA75/17-M-L239D). These mutations were chosen because they efficiently prevented M to bud from the cell, whereas all other functions remained unaffected. To successfully rescue budding-deficient viruses, we transiently coexpressed vesicular stomatitis virus (VSV)-G in Bsr-T7 cells, which contains the ability to drive cell entry of pseudotyped CDV virions in an F/H-independent manner and, most importantly, to produce VLPs on its own. Therefore, the latter function was employed to enable unspecific packaging of RNPs (regardless of the M gene) into pseudotyped CDV particles. Approximately 30 to 60 PFU of the G-complemented recombinant viruses was then transferred to Vero-cSLAM cells (Fig. 8C). The impact of the mutated M proteins on infectivity was assessed at 3 days postinfection by determining the 50% tissue culture infective dose (TCID_50_) per ml of infectious particles, either associated with the plasma membrane (CA) or free within the cell supernatant (SN).

Interestingly, both the CA and SN titers of rA75/17-M-E89R (3.2 × 10^4^ and 6.2 × 10^1^ TCID50/ml, respectively) were significantly reduced compared to rA75/17-M-wt (3.4 × 10^5^ and 2.6 × 10^3^, respectively) (Fig. 8A and B). Similar results were obtained with rA75/17-M-L239D, which exhibited even stronger deficiencies compared to rA75/17-M-E89R (Fig. 8A and B).

Overall, the reduced infectivity monitored with viruses harboring mutated M proteins strengthened the VLP data and further supported the essential role of both putative α-helices of CDV M in viral egress and ensuing infectivity.

### Mutations within both α2/9 microdomains generate M variants with impaired mobility selectively at the PM.

The data summarized above indicated that some specific M mutants (e.g., M-E89R or M-L239D) were affecting viral fitness by specifically interfering with the budding capacity. More specifically, the mutations introduced were designed to map within M microdomains potentially involved in M oligomer assembly (dimer-dimer contacts). Interestingly those mutants showed proper intracellular protein concentration, localization at the PM, interaction with N, and triggering of cell protrusions. However, their deficient budding activity suggested that the production of M networks might have been either prevented or impaired, thereby interfering with membrane budding activity.

To shed further light on the molecular mechanisms promoting M oligomer formation, we took advantage of the raster image correlation spectroscopy (RICS) method (46). RICS technology informs on the dynamics of a given protein in living cells, which is obtained from the stack of frames collected by time-lapse fluorescence microscopy. This methodology enables the calculation of a diffusion rate, which indirectly correlates with the size of a protein. If a large protein complex (e.g., an oligomer) is compared to its monomeric variant, or if the protein of interest interacts with any other cellular components, the diffusion rates will vary. This technique was successfully employed to study the oligomeric states of the related Ebola virus matrix (VP40) protein (47).

**FIG 9:**
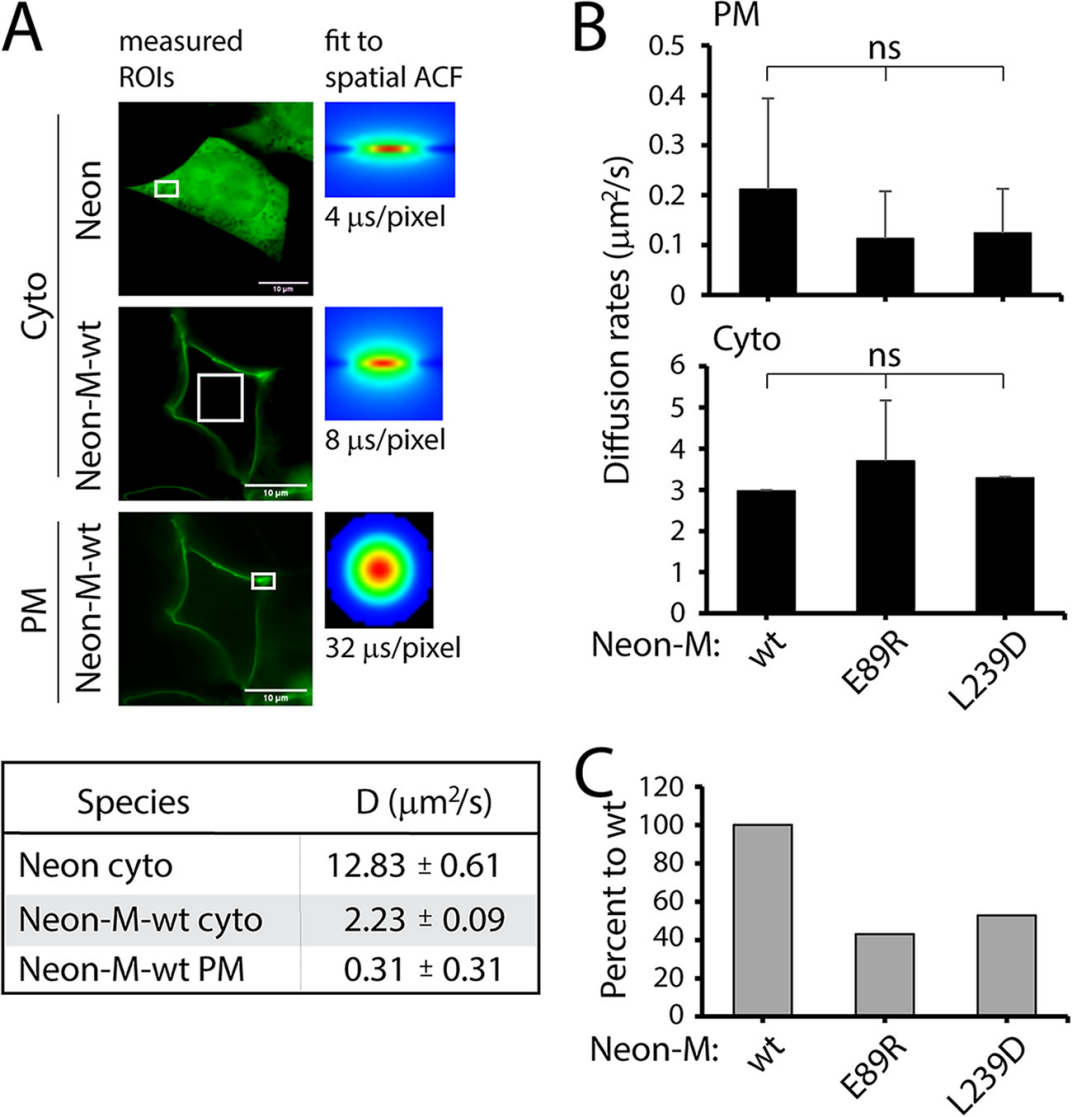
RICS analysis of wt and mutated M proteins in the cytosol and at the PM. (A, upper panels) Green fluorescence images recorded from Neon- and Neon-M-wt-expressing HEK-293T cells at 24 h posttransfection. The white box highlights the analyzed regions of interest (ROIs). The fit to spatial autocorrelation function (ACF) represents an average of 100 spatial ACFs from 100 images after background subtraction within the measured ROI in order to determine the diffusion coefficient. Scan speeds are indicated. (A, lower panel) Summary of recorded diffusion rates for Neon and Neon-M-wt. (B) Diffusion rates of Neon-M-wt and mutants recorded at the PM (upper panel) or in the cytosol (lower panel) at 48 h posttransfection. (C) Ratio of PM versus cytosolic diffusion rate values of Neon-M-wt and mutants (normalized to wt). Means and SD of data of a minimum of two independent experiments are shown. Statistical significance was determined using one-way ANOVA followed by Tukey test using GraphPad Prism 8 to analyze differences between wild-type and mutant means; ns, not significant.

Toward this aim, we initially fused a green fluorescent protein (mNeonGreen [Neon]) to the N-terminal domain of CDV M, with this construct maintaining full functionality (data not shown). We then introduced targeted α2/9 mutations (M-E89R and M-L239D) that selectively affected membrane budding activity. Compared to the cytosolic localization of Neon expressed alone, Neon-M-wt was unambiguously distributed to the PM (Fig. 9A, upper panels). The diffusion rates of Neon and Neon-M-wt within the cytosol were then calculated at 24 h posttransfection. As expected, the mobility of Neon in the cell was much faster than its derivative fusion construct (Neon-M-wt is about 40 kDa larger) (Fig. 9A, lower panel). Next, the Neon-M-wt diffusion rates were measured at the plasma membrane and in the cytosol. As expected for a protein that interacts with the inner leaflet of the PM (and may form oligomer at the PM), the diffusion rate of Neon-M-wt at the PM was about seven times slower than the one recorded in the cytosol (0.31 ± 0.31 μm^2^/s versus 2.23 ± 0.09 μm^2^/s) (Fig. 9A, lower panel). We noted that the values recorded at the PM were consistently more variable than those monitored in the cytosol, which could be attributed to more heterogeneous M oligomeric populations at this specific cellular location.

We then subjected both α2/9 M mutants (M-E89R and M-L239D) to RICS analysis at 48 h posttransfection. In this series of experiments, although not significant, the diffusion rate of M-wt at the PM had the tendency to be faster than the two M mutants, whereas values recorded in the cytosol were very similar for all M proteins (Fig. 9B). When the PM/cytosol ratio (normalized to M-wt) was calculated, it became apparent that both M mutants were characterized by substantially slower dynamics in the cell (Fig. 9C).

Taken together, these data first reveal that Neon-M-wt exhibited slower dynamics at the PM than within the cytosol. Furthermore, our findings revealed that introducing single mutations within the putative α2/9-helices of CDV M affects its mobility.

## DISCUSSION

A recent structural (cryoelectron tomography; cryo-ET) study reported that the matrix protein of MeV assembles into 2D “grid-like” structures under both the viral envelope and the plasma membrane of infected cells (45). Given that similar data were also reported for some related paramyxoviruses and pneumoviruses (i.e., Sendai virus, NDV, and RSV), the assembly of M networks underneath lipid bilayers may be an important common feature to those enveloped RNA viruses, and may be employed for cell exit. However, high-resolution structural detail of such M oligomers, as well as the mechanistic link between M array formation and viral budding activity, remain to be elucidated. This is critical, because providing such refined structural and functional knowledge on the viral egress process may translate into the development of attractive novel antiviral strategies.

Although providing important novel information, the limited resolution of the cryo-ET study of MeV M grids not only precluded the precise spatial positioning of M within the network, but could not identify microdomains essential to the promotion of M oligomer assembly. We and others have hypothesized that the angle formed at M dimer-dimer interfaces may contribute to membrane curvature, which in turn may promote membrane scission and viral egress (6, 41, 43). Interestingly, based on this assumption, candidate microdomains were proposed to be involved in M dimer-dimer interactions; one was a putative α-helix mapping within M-NTD (α2) and another one resided within M-CTD (α9), both microdomains locating opposite to the large dimeric interface (Fig. 1A and B). In this study, we aimed at investigating the potential role of both putative α-helices of CDV M in the promotion of membrane budding activity.

In a recent functional study, we identified several surface-exposed lysine residues located within a large basic patch of M-CTD that were likely involved in electrostatic membrane interaction. These data enabled us to propose a model contributing to explaining how M may be positioned toward the PM (48). Based on this model, the location of both helices may provide supporting ground for dimer-dimer contacts and consequent formation of a 2D-M array. To provide further mechanistic information, both microdomains (putatively folding into α2/9-helices) were randomly mutated using both conservative and nonconservative single point mutations, and the derivative M mutants were subjected to thorough biochemical, functional, virologic, and cellular analyses.

Remarkably, most of the engineered α2/9-helices M mutants (P87A, K92E, A94S, L96N, E89D, E89H, E89R, D235N, Y236F, L239D, K240Q, E242Q, and K243E) preserved essential functions, including proper expression, dimerization, PM localization, interaction with N, and triggering of membrane protrusions. This was rather unexpected, since most of the mutations introduced within the putative helices were nonconservative amino acid substitutions (expect for M-E89D and M-Y236F) and these microdomains are well conserved among morbilliviral members. Nevertheless, among these 13 single-point M mutants, only four of them preserved substantial budding efficiency (>70%: M-P87A, M-E89D, M-D235N, and M-E242Q), whereas all others exhibited clear defects in VLP formation and the production of infectious viruses (exemplified by rA75/17-M-E89R and M-L239D). Although impairment in viral infectivity may result from the production of noninfectious virions, our VLP data rather suggested that productive viral egress was the cause of the latter recorded defective infectivity. Collectively, this is the first time that rationally designed single-point M mutants corroborated with selective defects in late stages of the membrane budding process and ensuing viral infectivity. We hypothesized that the latter phenotype resulted from defective or irregular (i.e., nonproductive) M network assembly, thereby inferring an essential role of both targeted M microdomains (α2/9-helices) in controlling potent 2D-M array formation.

In good agreement with our hypothesis, the RICS analyses provided important additional information, strengthened by the technology employed, which enables monitoring of the dynamics of a given protein in the cell. Of major importance, if the protein of interest is involved in interactions with host factors (complex formation), with itself (oligomerization), or with any other cellular components (e.g., lipids), and this occurs in certain location of the cell only, variation of diffusion rates can be measured by RICS analysis. Interestingly, when diffusion rates of two selected M mutants (E89R and L239D) were recorded and compared to M-wt, a tendency for a reduced mobility at the PM was observed. This suggested that, while substituting the α2/9 microdomains of M may not significantly influence the M oligomeric state in the cytosol, it might impact the M oligomeric populations at the PM. Indeed, the varying populations of M (wt and mutants) at the cell periphery (perhaps highlighting the dynamic process of M oligomerization at this location) was consistent with finding that diffusion rates recorded at the PM were consistently more various than those monitored in the cytosol. In addition, such inherent variations in M mobility at the cell surface may also explain the lack of statistical differences.

These findings allowed us to speculate that the tested α2/9 M mutants might have folded into M dimers capable of reaching the PM and inducing membrane deformation, but were unable to form budding-competent networks. Consequently, such M dimers might have been trapped in lipid microdomains (perhaps facilitating interaction with irrelevant host factors) that in turn would have prevented the generation of productive oligomers. Alternatively, the reduced diffusion rates recorded with the mutated M proteins might have resulted from dimers assembling into irregular oligomers (generating only 1D oligomers or 2D but stiffer/larger M complexes) incompatible with proper VLP and/or progeny virus production. Nevertheless, although the precise molecular mechanism remains to be determined, our data unambiguously highlighted for the first time the major importance of two spatially discrete M microdomains in late stages of the viral life cycle.

Interestingly, a role of the conserved glutamic acid at position 89 (residing within the putative α2-helix) of the morbilliviral M protein was reported. While the E89G mutation occurred when wt MeV was adapted to grow in Vero cells (lacking natural receptors) (38), the E89K mutation is found in some MeV vaccine strains. Remarkably, both substitutions enabled MeV to infect Vero cells more efficiently (37, 38). Specifically for CDV M, the mutants E89G and E89Y were retained in the cytosol, pointing toward defects in intracellular transport competence. Such phenotypes evidently correlated with M protein variants lacking the capacity to trigger membrane deformation and ensuing budding activity. Additionally, the CDV M-E89K mutant was unable to potently bud VLPs. Therefore, although various molecular mechanisms may exist between different members of the genus *Morbillivirus*, these data taken together indicate that the negative charge of the amino acid residing at position 89 of the morbilliviral M protein, and locating within the putative α2-helix, contributes to viral egress. This assumption was further supported by the engineered M-E89D conservative mutant, which exhibited wt-like functions.

It is important to note, that, although both M-E89R and M-L239D mutants displayed similar defects in VLP production, recombinant viruses harboring the M-L239D mutation exhibited more prominent impairments in the production of infectious progeny viruses. We hypothesized that while only unproductive M oligomers might have been induced by the sole expression of either M-E89R or M-L239D, when folding in the presence of all other viral components, some compensational mechanisms might have partly restored productive M array assembly (and hence the ensuing viral budding activity) in the case of rA75/17-M-E89R. Accordingly, these findings spotlighted the pivotal role of the α9 M microdomain in viral-induced membrane budding activity and ensuing infectivity, perhaps by acting as a more dominant coordinating domain in the assembly of high-order oligomers.

In summary, by introducing single point mutations in two microdomains of CDV M, proposed to be involved in dimer-dimer contacts, we engineered some M protein variants that were selectively impaired in late stages of membrane budding activity, whereas other functions (including expression/stability, folding, intracellular transport competence, plasma membrane deformation, and interaction with N) remained overall unaltered. Because such M mutants had the tendency to exhibit a reduced mobility specifically at the PM (compared to their dynamics within the cytosol), our data were suggestive of a role of both M microdomains in controlling proper high-order oligomer formation. In turn, proper 2D-M array formation at the PM, partly controlled by two discrete microdomains (α-helices), may contribute to efficient morbilliviral-induced membrane scission and budding activities.

## MATERIALS AND METHODS

### Cell cultures, plasmid construction, and transfections.

HEK-293T (human embryonic kidney-293T cells; ATCC CRL-11268), Bsr-T7 (baby hamster kidney 21 [BHK-21]) cells constitutively expressing T7 RNA polymerase (49), and Vero-cSLAM (African green monkey kidney epithelial cells stably expressing canine SLAM receptor, kindly provided by Yusuke Yanagi, Kyushu University, Japan) cells were grown in Dulbecco’s modified Eagle medium (DMEM; Gibco, Life Technologies) supplemented with 10% fetal calf serum (FCS; BioSwissTech) and 1% penicillin-streptomycin (P/S) at 37°C in the presence of 5% CO_2_. Amplification of the matrix protein gene from a plasmid coding for the full-length antigenome of the CDV A75/17 strain was performed with supplementary RsrII restriction sites, N-terminal tags, and a linker (i.e., FLAG-linker-M). Using the restriction endonuclease RsrII, amplicons were subcloned into the vector pCI. All mutations to replace the FLAG tag with an HA or mNeonGreen (Neon) tag, to elongate the HA tag or the linker (2×HA-longlinker-M), and to replace the amino acids within the α-helices were performed in the pCI-FLAG-linker-M construct by using an In-Fusion HD cloning kit (TaKaRa Bio Europe) according to the manufacturer’s instructions. HA-linker-N_CDV_ was generated following the same strategy. The cells were transfected at 80 to 90% confluence with the various vector constructs using TransIT-LT1 (Mirus) reagent following the supplier’s instructions.

### Western blotting.

HEK-293T cells expressing various pCI constructs were harvested in 1× phosphate-buffered saline (PBS) at 24 h or 48 h posttransfection and centrifuged (10 min, 2,000 × *g*, 4°C). Cell pellets were resuspended in Laemmli buffer (2×) (Sigma-Aldrich), boiled at 98°C for 10 min, and loaded onto 12% Tris-glycine SDS-PAGE gels. Western blot analyses were performed using a primary antibody of either an in-house-generated rabbit polyclonal anti-CDV M antibody (αM; 1:1,000 in 5% milk) (6) or a mouse monoclonal anti-tubulin (αTub; AbCam, 1:5,000). A horseradish peroxidase (HRP)-conjugated goat anti-rabbit or HRP-conjugated goat anti-mouse (Dako, 1:5,000) antibodies were used as secondary antibodies. Signals were detected on a nitrocellulose membrane (GE Healthcare Amersham Protran Premium 0.2 μm NC nitrocellulose membranes, Fisher) using a chemical colorimetric substrate (1-Step Ultra TMB-blotting solution, Thermo Fisher Scientific) following the manufacturer’s instructions. Anti-CDV M and anti-tubulin signal intensities from three independent experiments were quantified using the FUSION FX software (VILBER). The signal intensity ratio between the VLP production and the construct’s expression in total cell lysate (VLP/TL) was calculated and normalized to the wild-type (wt), which was set at 100%. The expression level was calculated by dividing the intracellular protein concentration of the construct in total cell lysate (TL) by the loading control (tubulin; Tub) and normalizing to the reference or the wt, which was set at 100%. For each mutant, the mean ratio of three independent tests is shown.

### Coimmunopurification.

FLAG-M and 2×HA-longlinker-M constructs, both bearing the same mutations, were cotransfected in HEK-293T cells seeded in six-well culture plates. At 24 h posttransfection, the cells were resuspended in PBS and centrifuged (10 min, 2,500 × *g*, 4°C). After removing the PBS, the cell pellets were lysed in lysis buffer (50 mM Tris [pH 7.4], 150 mM NaCl, 0.05% NP-40, 5% glycerol, 20 mM sodium molybtate) containing protease inhibitor (Roche, cOmplete mix) for 30 min at 4°C. Lysates were cleared (16,100 × *g*, 20 min, 4°C) and Laemmli buffer (2×) was directly added to 40 μl of the supernatant and boiled at 98°C for 10 min for the total cell lysates (TL).The remainder of the supernatant was incubated (at 4°C) for 1 h with a mouse monoclonal anti-HA antibody (Anti-HA.11 Epitope tag clone 16B12, LucernaChem) and overnight with 15 μl of prewashed Dynabeads Protein G (Novex; Life Technologies) on a rotating wheel. After three washes with 1× PBS-Tween20 (PBS-T), the bead samples were resuspended in 40 μl of Laemmli buffer (2×) and boiled at 98°C for 10 min precisely. Supernatants were then subjected to Western blot analyses using a rabbit polyclonal anti-CDV M as the primary antibody (1:1,000) (6), as described above, for at least 1 h at room temperature. Expression or coimmunopurification of FLAG-M and 2×HA-longlinker-M anti-M bands (coimmunopurified protein_coIP and immunopurified protein_IP, respectively) intensities were determined simultaneously for each mutant, using the FUSION FX software (VILBER). The coIP efficiency index was determined as the ratio of coIP to IP signals (FLAG-M/2×HA-longlinker-M) divided by the ratio of total cell lysates (FLAG-M/2×HA-longlinker-M) normalized to wt, which was arbitrarily set at 100%. For each mutant, the mean index of three independent experiments is shown.

### Indirect immunofluorescence analyses.

Twenty-four-well cell culture plates were covered with glass coverslips, specifically for indirect immunofluorescence and confocal microscopy assays, and used for seeding of HEK-293T cells. These cells, transfected with plasmids encoding M or derivative M mutant proteins, were fixed with 4% paraformaldehyde at 24 h posttransfection and then permeabilized using 0.5% Triton X-100 (10 to 15 min at room temperature for each step). Analyses were performed using a mouse monoclonal anti-FLAG M2 antibody (1:1,000, Sigma-Aldrich) for FLAG-M constructs, a rabbit monoclonal anti-sodium-potassium ATPase (anti-Na/K ATPase) antibody (1:600, Abcam) for the plasma membrane, and a rat monoclonal anti-HA antibody (1:1,000, Roche Diagnostics) for the HA-N construct as primary antibodies. Alexa Fluor 555-conjugated goat anti-mouse IgG (H+L), Alexa Fluor 488-conjugated goat anti-rabbit IgG (H+L), Alexa Fluor 488-conjugated goat anti-mouse IgG (H+L), and Alexa Fluor 555-conjugated goat anti-rat IgG (H+L) (1:1,000; Life Technologies) were used as secondary antibodies. Nuclei were stained with 4’,6-diamidino-2-phenylindole (DAPI; 1:5,000, 1 μg/ml, AppliChem). Pictures were acquired by laser scanning confocal microscopy using an Olympus FV3000 confocal microscope with Olympus FV3000 RS Fluoview software and analyses of the pictures, as well as three-dimensional (3D) reconstitutions, were done with Imaris software.

### Virus-like particles assay.

HEK-293T cells, seeded in 6-well culture plates, were transfected with plasmids expressing different FLAG-M constructs (3 μg). Culture supernatants and cells were collected at 48 h posttransfection. Cellular debris was removed from supernatants by centrifugation (20 min, 500 × *g*, 4°C). Clarified supernatants were incubated for 30 min at 37°C with 40 μl of trypsin (trypsin-EDTA solution [10×]; Sigma-Aldrich). Subsequently, 50 μl of soybean trypsin inhibitor (1 mg/ml dissolved in distilled water; Roche Diagnostics) was added to neutralize the protease. The resulting mixture was loaded onto a 25% glycerol cushion and centrifuged at 16,100 × *g* for at least 1 h at 4°C. The pellet was resuspended in Laemmli buffer (2×), boiled at 98°C for 10 min, and then subjected to Western blot analyses as described above, using a rabbit polyclonal anti-CDV M antibody (1:1,000). As defined previously, cells were resuspended in 1 ml PBS (1×) and centrifuged (2,000 × *g*, 10 min, 4°C). Cell pellets were lysed in lysis buffer for 30 min at 4°C before being cleared (16,100 × *g*, 20 min, 4°C). Laemmli buffer (2×) was added into lysate supernatants and then boiled at 98°C for 10 min before being subjected to Western blot analyses. As mentioned before, and as we wanted to assess the intrinsic capacity to produce VLPs, the signal intensity ratio between the VLP production and the construct’s intracellular protein concentration (VLP/TL) was calculated and normalized to the wt. Each mutant’s efficiency index for VLP production was calculated relative to the wt index (arbitrarily set to 100%) and, for each mutant, the mean ratio of three independent experiments is shown.

### Generation of recombinant viruses.

Rescue of mutated recombinant viruses were conducted by initially transfecting Bsr-T7 cells seeded in 6-well plates with the plasmid encoding the A75/17 antigenome (harboring the mutation in an M gene) together with helper (pTM-N, P and L [48]) plasmids, as well as pMD2.G (addgene), which encoded the VSV-G glycoprotein. The latter was added in order to restore viral budding in case of defective M-carrying recombinant viruses. Transfections were performed using the TransIT-LT1 reagent (Mirus), according to the manufacturers' instructions. At 3 h posttransfection, the cells were heat shocked for 2 h at 42°C, the medium was exchanged, and cells were further incubated for 48 h at 37°C. Finally, cells and supernatants were harvested, submitted to one cycle of freezing and thawing, and centrifuged (5 min at 800 × *g*, 4°C). The collected supernatants were then used to inoculate 6-well plates previously seeded with 0.7 × 10^6^ Vero-cSLAM cells. Three days postinfection, cell-associated (CA) and cell-free (SN) viruses were collected separately and submitted to titration experiments. To this aim, the 50% tissue culture infective dose (TCID_50_) was calculated according to the Spearman & Kärber method (50, 51).

### RICS data acquisition.

Raster image correlation spectroscopy (RICS) is the ideal method to measure molecular dynamics, such as diffusion coefficients, based on fluorescence confocal images. By measuring changes in diffusion coefficients, RICS can indirectly detect protein binding. The scanning movement of any confocal microscope creates a space-time matrix of pixels within each image. The time between scan lines and the time between images represent the spatial and temporal sampling time of the laser beam (pixel dwell time). For RICS analysis, individual frames of a time series are spatially correlated and the average of all frames is taken. Immobile structures and slowly moving features must be removed by background subtraction. Accordingly, RICS allows detection of differences in protein mobility via measuring diffusion rates in different regions of interest (ROIs) within an image. HEK-293T cells were seeded in μ-Dish 35 mm, high (Ibidi) and transiently transfected with plasmid encoding mNeonGreen (Neon) fluorescent protein alone or mNeonGreen tagged M protein (Neon-M-wt). RICS measurements were performed at 24 h or 48 h posttransfection. RICS data were acquired on a commercial laser scanning confocal microscope (Zeiss LSM880 inverted microscope) using a Zeiss Plan Apochromat 63×/1.2 water objective. The 488 nm line of the Argon laser was used for excitation of mNeonGreen protein. The emission from 493 nm to 556 nm was collected with a GaAsp detector. The data were collected as images of 256 × 256 pixels with a pixel size of 50 nm for 100 frames of the image series. The scan speed (or pixel dwell time) ranged from 4 to 32 μs/pixel, depending on the diffusion rate of the molecule. RICS analysis was done using the RICS module of Zen black edition 2.3 (Zeiss).
